# Evaluation of *Galleria mellonella* larvae for studying the virulence of *Streptococcus suis*

**DOI:** 10.1186/s12866-016-0905-2

**Published:** 2016-12-15

**Authors:** Nadya Velikova, Kevin Kavanagh, Jerry M. Wells

**Affiliations:** 1 Host-microbe Interactomics Group, Department of Animal Sciences, Wageningen University, Zodiac 122, De Elst 1, 6708WD Wageningen, The Netherlands; 2 Department of Biology, Maynooth University, Co. Kildare, Ireland

**Keywords:** *Streptococcus suis*, *Galleria mellonella*, Virulence, Reduction and refinement (3Rs), Infection model

## Abstract

**Background:**

*Streptococcus suis* is an encapsulated Gram-positive bacterium and the leading cause of sepsis and meningitis in young pigs, resulting in considerable economic losses in the porcine industry. *S. suis* is considered an emerging zoonotic agent with increasing numbers of human cases over the last years. In the environment, both avirulent and virulent strains occur in pigs, with no evidence for consistent adapatation of virulent strains to the human host. Currently, there is an urgent need for a convenient, reliable and standardised animal model to rapidly assess *S. suis* virulence. Wax moth (*Galleria mellonella*) larvae have successfully been used in human and animal infectious disease studies. Here, we developed *G. mellonella* larvae as a model to assess virulence of *S. suis* strains.

**Results:**

Fourteen isolates of *S. suis* belonging to different serotypes killed *G. mellonella* larvae in a dose-dependent manner. Larvae infected with the virulent serotype 2 strain, *S. suis* S3881/S10, were rescued by antibiotic therapy. Crucially, the observed virulence of the different serotypes and mutants was in agreement with virulence observed in piglets (*Sus scrofa*) and the zebrafish larval infection model. Infection with heat-inactivated bacteria or bacteria-free culture supernatants showed that in most cases live bacteria are needed to cause mortality in *G. mellonella*.

**Conclusions:**

The *G. mellonella* model is simple, cost-efficient, and raises less ethical issues than experiments on vertebrates and reduces infrastructure requirements. Furthermore, it allows experiments to be performed at the host temperature (37 °C). The results reported here, indicate that the *G. mellonella* model may aid our understanding of veterinary microbial pathogens such as the emerging zoonotic pathogen *S. suis* and generate hypotheses for testing in the target animal host. Ultimately, this might lead to the timely introduction of new effective remedies for infectious diseases. Last but not least, use of the *G. mellonella* infection model to study *S. suis* virulence adheres to the principles of replacement, reduction and refinement (3Rs) and can potentially reduce the number of vertebrates used for experimental infection studies.

**Electronic supplementary material:**

The online version of this article (doi:10.1186/s12866-016-0905-2) contains supplementary material, which is available to authorized users.

## Background

*Streptococcus suis* is an emerging zoonotic Gram-positive bacterial pathogen [1, 2] and the leading cause of sepsis and meningitis in piglets. Infections with *S. suis* have been reported worldwide throughout the pig production industry and have led to considerable economic losses [3, 4]. Recently, *S. suis* isolates causing more rapid and severe infections in both humans and pigs have been reported [5], raising concerns about the emergence of more virulent strains [6–10].

The most common symptoms caused by virulent *S. suis* in pigs and humans are meningitis, endocarditis and streptococcal toxic shock-like syndrome [11–13]. In spite of the importance of *S. suis* as a porcine pathogen and zoonotic agent, relatively little is known about epidemiology and virulence mechanisms leading to carriage and invasive disease. Genetic analysis of virulence and pathogenicity is not trivial as *S. suis* possesses multifactorial virulence factors [1]. The matter is further complicated because natural populations are characterized by high rates of recombination [14, 15], leading to many different genotypes [4]. A generally accepted virulence factor of *S. suis* is the capsular polysaccharide [16]. The critical role of the capsular polysaccharide in virulence and pathogenicity of *S. suis* has been demonstrated in multiple independent studies [17–19]. Of the 33 serotypes of the capsular polysaccharide identified so far, serotype 2 is the most frequently associated with invasive disease [6, 20]. Other serotypes often associated with porcine disease include serotype 1, 9, and 14 [21, 22]. *S. suis* colonises the upper respiratory tract of pigs and the intestine [23, 24]. Healthy pigs carrying *S. suis* are a source for *S. suis* transmission to the rest of the pigs in the herd [23]. Currently, it is not possible to distinguish a virulent strain from a non-pathogenic strain using molecular markers [1] highlighting the need for better genomic markers of virulence and/or animal models to rapidly assess the virulence of different *S. suis* isolates.

Pigs and mice have been successfully employed for *S. suis* virulence studies [5, 25]. However, pigs and mice have economical, logistic and ethical disadvantages over non-mammalian infection models. Recently, the zebrafish (*Danio rerio*) larval model has been validated for assessing virulence of *S. suis* strains [26]. *G. mellonella* larvae have been successfully utilised to study the virulence of other important pathogens, including *Streptococcus spp.*, and to characterize existing and novel antibacterials [27–32]. *G. mellonella* larvae are exempt from ethical legislation. The larvae are cheap to purchase allowing experiments to be performed with a large number of samples. Furthermore, unlike other non-mammalian models, e.g. *Caenorabditis elegans*, *Drosophila melanogaster*, or zebrafish larvae, *G. mellonella* allows experiments to be performed at 37 °C, the body temperature of the natural hosts. Last but not least, the insect immune system exhibits both humoral and cellular components, and in some aspects the immune response is similar to the innate immune response of mammals [33]. For example, both insect hemocytes and mammalian neutrophils engulf and kill pathogens, and both types of cell produce superoxide, using similar p47 and p67 proteins [34]. Nevertheless, *G. mellonella* larvae are distantly related to pigs, and it is important to establish their predictive value for virulence in the natural host. A first step towards this goal is to compare the virulence of a range of clinical and non-clinical *S. suis* strains that differ in virulence in the natural host. Here, we evaluated the use of *G. mellonella* larvae as an in vivo model to assess virulence of *S. suis* isolates.

## Methods

### Bacteria and growth conditions

*S. suis* strains used in this study are listed in Table 1. *S. suis* were stored at −80 °C in 20% (v/v) glycerol. *S. suis* strains were cultured routinely in Mueller Hinton broth (MHB) (Difco, USA) at 37 °C in the presence of 5% CO_2_ without shaking. For each strain studied, a calibration curve was generated for absorbance measurements of bacterial cultures at 600 nm (OD600) by plating aliquots on MH agar (1% v/v) to calculate the bacterial colony forming units per milliliter (CFU/ml) (data not shown).

**Table 1:** Virulence of bacterial strains used in this study. HV stands for highly virulent, V stands for virulent, WV stands for weakly virulent, and AV stands for avirulent. The virulence in pigs is described in [35] and the scale of virulence in pig infection model is as described in [35]. Strains leading to more than 50% mortality of *G. mellonella* larvae post-infection with 10^7^ CFU/ml were considered virulent, and strains leading to less than 50% mortality of *G. mellonella* larvae were considered weakly virulent

Strain	Serotype	Virulence in pigs [35]	Virulence in *G. mellonella*	Clinical source	Reference/source
6388	1	HV	V	Organs	Laboratory collection [45]
6555/NCTC 428	1	V	WV		
T15	2	AV	WV	Tonsil	Laboratory collection [46]
S735R2	2	WV	WV	Unknown	Laboratory collection [35]
P1/7	2	V	V	Unknown	Laboratory collection [35]
3881/S10	2	V	V		[47]
(J28) S10 cpsΔEF	2	AV	WV		[17]
15965	3	ND	V		
5213	4	ND	V		
8039	7	ND	V	CNS	Laboratory collection [40]
7709	9	ND	V	Bacteraemia	Laboratory collection [40]
C132	9	ND	WV	Brain/septicemia	Laboratory collection [40]
7998	9	ND	V	Joint	Laboratory collection [40]
8186	9	ND	WV	Tonsil	Laboratory collection [40]
5128/22083	9	ND	V		

*V* virulent, *HV* highly virulent, *WV* weakly virulent, *AV* avirulent, *ND* not determined

### *G. mellonella* larvae

*G. mellonella* larvae in their final instar stage were purchased (UK Waxworms Ltd, Sheffield, UK), and stored in the dark at 15 °C, and used within 14 days. Larvae were separated by weight, and only larvae between 0.2 and 0.3 g were used for experiments. Unless otherwise stated, all experiments in *G. mellonella* were performed using 15 larvae per group and in most cases repeated using larvae from a different batch to give *n* = 30. Larvae were injected with 20 μL of bacterial suspension, antibiotic solution, or phosphate buffered saline (PBS) in the left posterior proleg using Terumo Myjector 29G insulin syringes (VWR International). The syringes were changed between treatments with different strains. Two negative control groups were included in every experiment; one group was not injected to control for background larval mortality (no manipulation control) and the other group (uninfected control) was injected with PBS to control for the possible effect of physical trauma on mortality. Larvae were stored in Petri dishes in the dark at 37 °C with 5% CO_2_ for up to 144 h post-infection (p.i.) and inspected every 24 h for survival; larvae were considered dead if they did not move after shaking of the petri dish*.*

### Virulence of *S. suis *in *G.mellonella*

Infection of *G. mellonalla* larvae with *S. suis* strains was performed as described previously for other pathogens [27, 28]. Larvae were infected in the left posterior proleg with 20 μl inocula of different *S. suis* strains containing between 1 × 10^5^ CFU/mL to 1 × 10^8^ CFU/mL. Survival of larvae was recorded at 24 h intervals for 144 h p.i.. A heat-killed *S. suis* inoculum of  > 1 × 10^8^ CFU/ml incubated at 99 °C for 30 min was used as a negative control in the infection experiments. In some experiments *S. suis* culture supernatants were prepared by centrifugation of overnight cultures (i.e. > 1 × 10^8^ CFU/ml) at 11 000 g for 30 min and injected into the posterior proleg of groups of larvae as described above. The absence of viable *S. suis* in culture supernatants and heat inactivated cultures was confirmed by plating on MH agar and incubation at 37 °C for 24 h.

### Antibiotic treatment

Antibiotic stock solutions were prepared in 1× PBS. For studies of antibiotic efficacy, an inoculum of 1 × 10^7^ CFU/mL of *S. suis* 3881/S10 was used. A single treatment with antibiotics was administered 2 h p.i. via the posterior proleg.

### Statistical analysis

Survival data were plotted using the Kaplan–Meier method and comparisons between groups were made using the log-rank test using GraphPad Prism v 5.03. In all comparisons with the negative control, it was the uninfected control (rather than the un-manipulated control) that was used. In all tests, *P* ≤ 0.05 was considered significant. The lethal dose leading to 50% mortality (LD50) at different time points was determined by plotting the percentage of surviving larvae following infection with different inocula, and non-linear curve fitting using GraphPad Prism v 5.03.

## Results

### *Galleria mellonella * larvae are sensitive to infection by *Streptococcus suis*

**Fig. 1:**
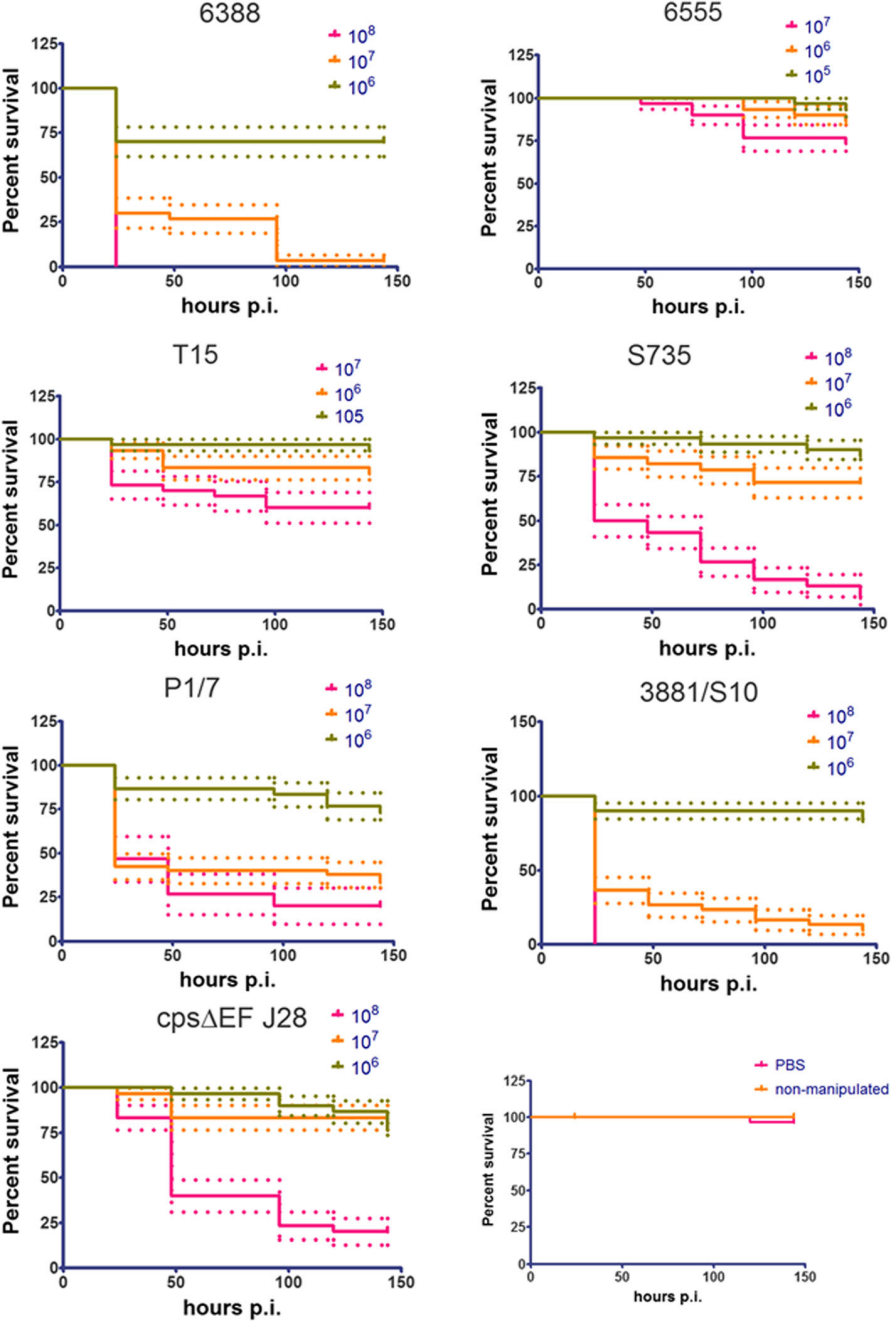
Dose dependent virulence of different *S. suis* strains belonging to serotype 1 to 3 (Table 1). Survival data were plotted using the Kaplan–Meier method using GraphPad Prism v 5.03. The dotted lines represent the standard error. The observed virulence is in agreement with virulence observed in a piglet infection model (Table 1)

**Fig. 2:**
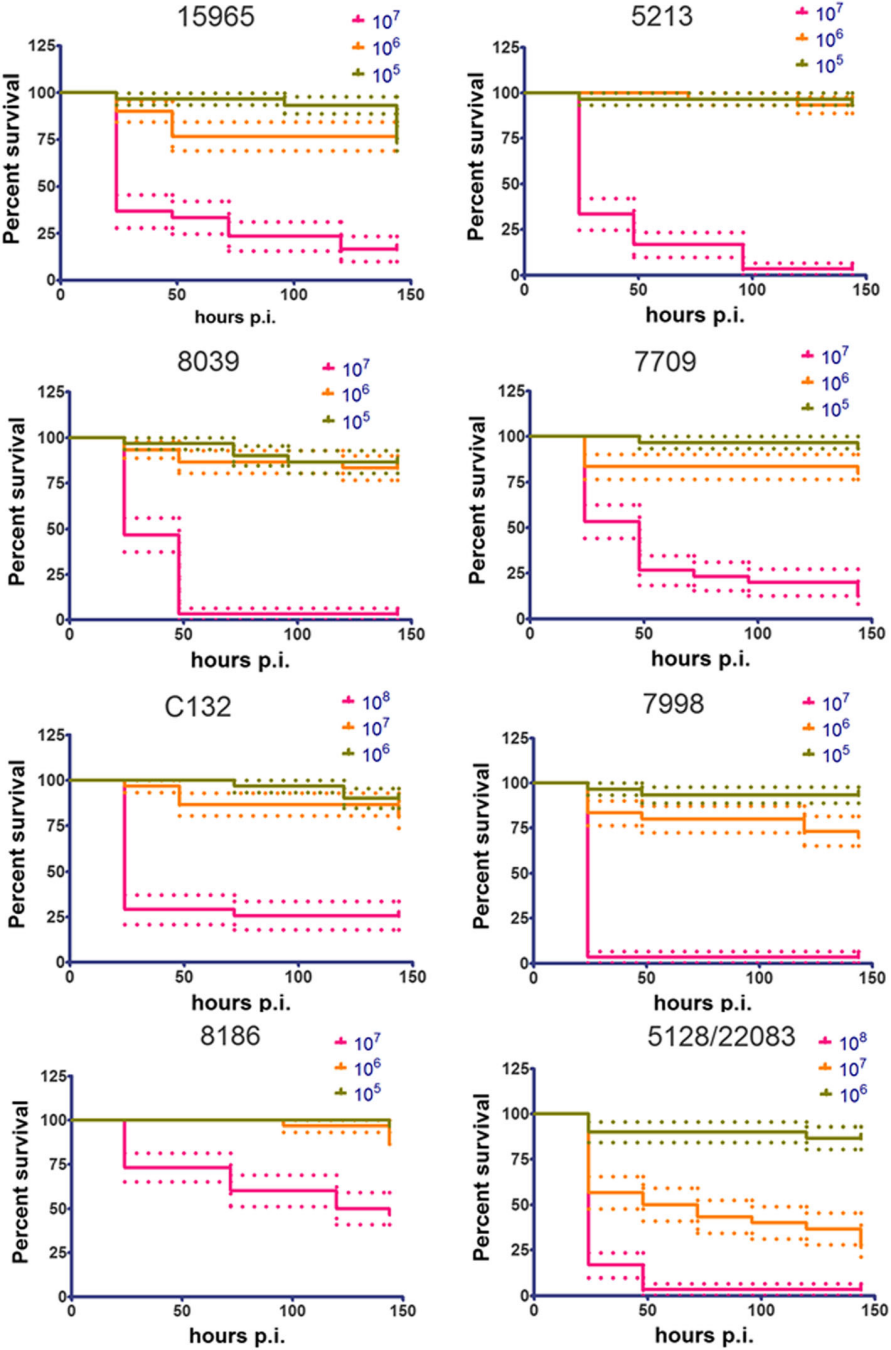
Dose dependent virulence of different *S. suis* strains belonging to serotype 4, 7 and 9 (Table 1). Survival data were plotted using the Kaplan–Meier method using GraphPad Prism v 5.03. The dotted lines represent the standard error. The observed virulence is in agreement with virulence observed in a piglet infection model (Table 1)

The virulence of *S. suis* isolates (Table 1) that had been assessed for virulence in a piglet infection model [35] was assessed in *G. mellonella* larvae by inoculation with 20 μL of suspensions containing 1 × 10^5^ to 1 × 10^8^ CFU/ml and incubation at 37 °C. Notably, isolates reported as virulent in pigs (6388, 6555/NCTC 428, P1/7, 3881/S10, 7997) [35] and in a zebrafish larval model (3881/S10, 6555/NCTC 428) [26] showed dose-dependent virulence in *G. mellonella* larvae (Figs. 1 and 2, Table 1). Isolates reported as weakly virulent or avirulent in piglets (T15, S735R2) showed attenuated virulence in *G. mellonella* larvae compared to the virulent strains, e.g. 3881/S10 (Figs. 1 and 2, Table 1). One generally accepted virulence factor of *S. suis* is the capsular polysaccharide, and its critical role in virulence and pathogenicity has been demonstrated in multiple independent studies [17, 18, 26]. A mutant lacking the capsular polysaccharide, the serotype 2 mutant cpsΔEF [17], showed more than 20 times attenuated virulence to *G. mellonella* larvae compared to the wild type, 3881/S10 (Figs. 1 and 2, Additional file 1: Figure S1, and Table S1). After 24 h p.i. 5 × 10^6^ CFU/ml of the wt 3881/S10 lead to 50% mortality, whereas more than 1×10^8^ CFU/ml of the mutant lacking the capsular polysacharyde, cpsΔEF, are needed for 50% mortality 24 h p.i. These results confirm the applicability of the *G. mellonella* larvae infection model to study *S. suis* virulence factors. Furthermore, we studied isolates for which virulence data in piglet infection models has not been reported. *G. mellonella* larvae were infected with isolates from different clinical sources (central nervous system, bacteraemia, speticemia, joins, and tonsils, Table 1). Six of the studied isolates (15965, 5213, 8039, 7709, 7998, 5128/22083) showed dose-dependent virulence (Figs. 1 and 2). The serotype 9 isolates 5128/22083 and 7709 were among the most virulent isolates tested in *G. mellonella* larvae (Figs. 1 and 2 and Additional file 1: Figure S1, and Table 1 and Additional file 1: Table S1). Another serotype 9 isolate, 7998, showed virulence comparable to the virulence of the serotype 2 isolates 3881/S10 and P1/7. Isolates 5213 (serotype 4) and 8039 (serotype 7) showed weaker virulence, comparable to the non-capsulated serotype 2 mutant cpsΔEF (Figs. 1 and 2 and Additional file 1: Figure S1 and Table 1 and Additional file 1: Table S1). The serotype 3 isolate 15965 showed virulence comparable to the virulence of P1/7 (serotype 2). Isolates C132 and 8186 (both serotype 9) were weakly virulent (Figs. 1 and 2 and Additional file 1: Figure S1, and Table 1 and Additional file 1: Table S1).

Melanisation in *G. mellonella* is thought to be a key part of the defence against a range of pathogens [29]. Melanin is deposited around microbes within the hemolymph where it is believed to facilitate pathogen killing [29]. Over the course of *S. suis* infection, *G. mellonella* larvae showed obvious signs of melanisation similar to melanisation induced by infection with other pathogens (Additional file 1: Figure S2).

### The culture supernatants or heat-treated extracts of specific strains induced mortality in *G. mellonella* larvae

To examine the effect of bacteria-free culture supernatants on larval survival, 20 μl was injected through the left posterior proleg. For most strains the supernatant of an overnight culture (more than 1 × 10^8^ CFU/ml) did not significantly affect larval survival (Additional file 1: Figure S3) indicating that the exotoxin suilysin or endotoxins released into the growth medium were not causing mortality. The exception was strain 7709 for which the bacteria-free supernatant showed significantly increased mortality compared to the PBS control (*p* value 0.0085) indicating toxicity due to secreted factor(s). The heat inactivated inoculum (containing both the heat-treated cells and heat-treated supernatant) of 7709 did not lead to a significantly different effect than the PBS control. These indicate that the factor(s) in the 7709 supernatant causing mortality of the *G. mellonella* larvae is temperature sensitive.

The heat-inactivated inoculum of 8039 and 5213 significantly increased larval mortality compared to the PBS injected control group (*p* values of 0.0481 and < 0.0001, respectively). The virulence of the live bacteria of 8039 and 5213 is comparable to the non-capsulated serotype 2 mutant cpsΔEF and to the weakly virulent serotype 2 isolate S735 (Figs. 1 and 2 and Additional file 1: Figure S1, and Table 1 and Additional file 1: Table S1).

### Antibiotic treatment rescues larvae infected with *S. suis* 3881/S10

*G. mellenella* larvae have been used to study antibiotic treatment for different bacterial and fungal infections [27, 35, 36]. To assess the utility of *G. mellonella* larvae to study antibacterial therapies against *S. suis*, we confirmed that antibiotic therapy could rescue larvae from *S. suis* infection. Larvae were infected with 1 × 10^7^ CFU of the *S. suis* strain 3881/S10, a well characterised serotype 2 strain previously shown to be virulent in pigs and used in our previous research [37, 38, 39]. At 2 h p.i. the larvae were treated with doxycycline, amoxicillin and ampicillin (25 mg/kg). Additional control groups were assessed for the toxicity of the antibiotic treatment (data not shown). Treatment with 25 mg/kg of each of the antibiotics rescued the larvae, and 144 h p.i. there was 100% survival rate of the larvae treated with the three different antibiotics, compared to infected larvae that were not treated with antibiotics (data not shown).

### Deletion of the two-component system CiaRH reduced *S. suis* virulence in *G. mellonella* larvae

**Fig. 3:**
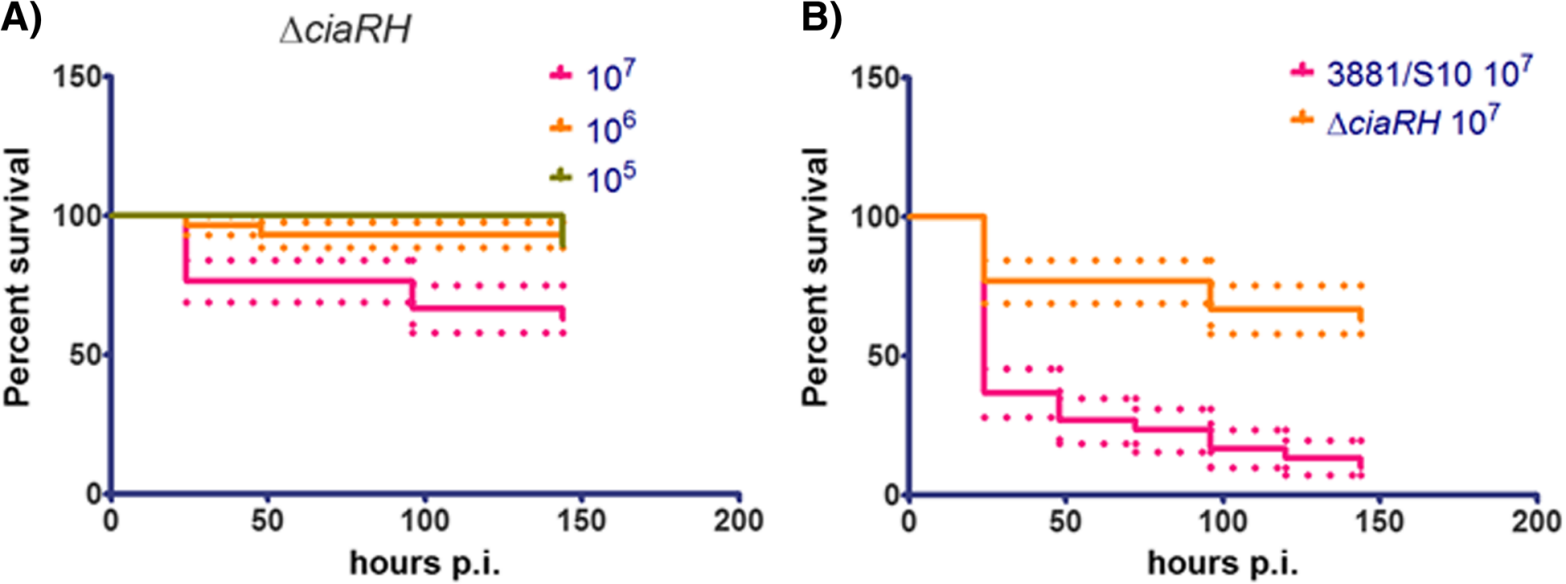
**a** Survival rate of *G. mellonela* larvae injected with 10^5^ – 10^7^ CFU/ml of the S10 Δ*ciaRH* mutant. **b** Comparison of the survival rate of larvae infected with 10^7^ of S10 Δ*ciaRH* mutant and the wild-type 3881/S10 strain. Survival data were plotted using the Kaplan–Meier method and comparisons between groups were made using the log-rank test using GraphPad Prism v 5.03. The dotted lines represent standard error

The two component system (TCS) CiaRH contributes to the virulence of *S. suis* serotype 2 [40]. Deletion of the *ciaRH* operon results in a lower survival rate in a bactericidal assay compared to the wild-type. Furthermore, the mutant showed reduced virulence in mice and pigs in vivo, and in a zebrafish larval model of *S. suis* infection [26]. In order to assess whether reduced virulence of *S. suis* strain S10 ΔciaRH would also be observed in *G. mellonella* larvae, we compared the survival of larvae infected with 1 × 10^7^ CFU/ml of the mutant strain S10 ΔciaRH or the wild type 3881/S10. Infection of larvae by the Δ*ciaRH* strain resulted in a significantly higher survival rate compared to infection with the 3881/S10 wild-type strain, (*p* < 0.0001) (Fig. 3). Thus, the observed results in *G. mellonella* larvae are in agreement with results obtained with the same two isolates in mouse and pig infection studies, and in zebrafish larval infection model. Taken together these results indicate that *G. mellonella* larvae can be used to assess virulence of porcine *S. suis* strains reproducibly and in a biologically meaningful way.

### *S. suis* suilysin mutant shows reduced virulence in *G. mellonella* larvae

**Fig. 4:**
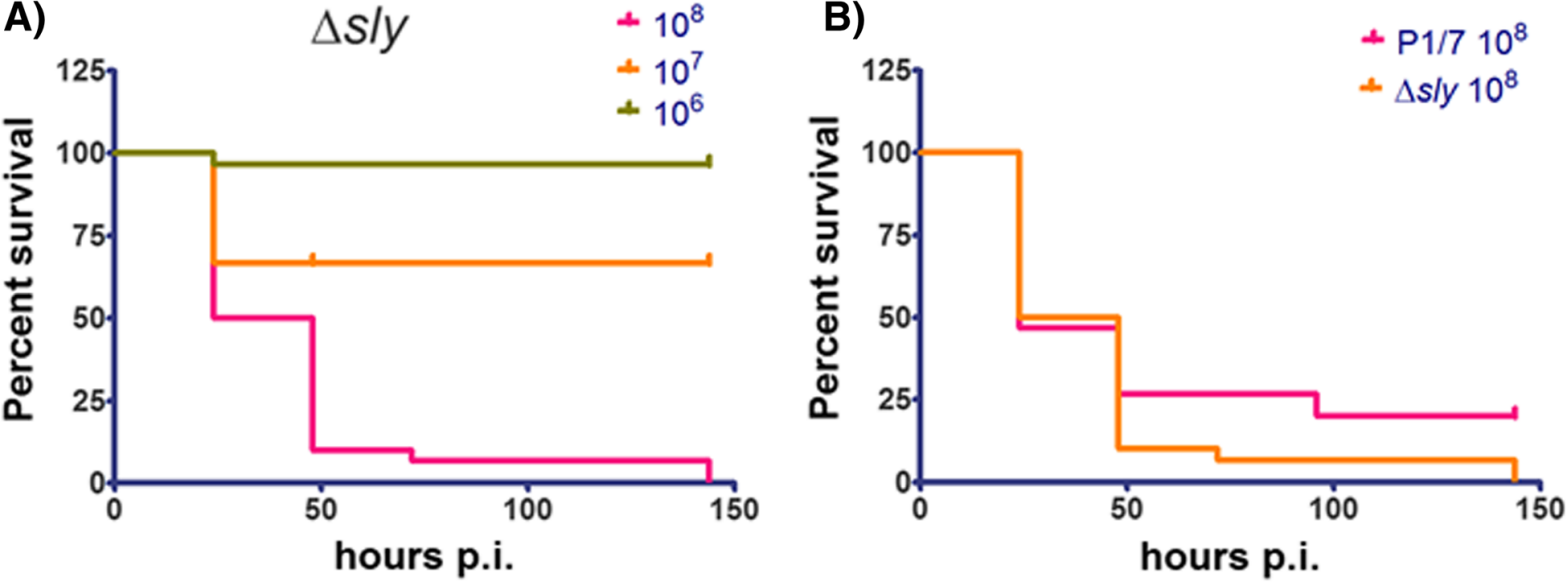
**a** Survival rate of *G. mellonela* larvae injected with 10^6^ – 10^8^ CFU/ml of the P1/7 Δ*sly* mutant. **b** Comparison of the survival rate of larvae infected with 10^7^ of P1/7 Δ*sly* mutant and the wild-type P1/7. Survival data were plotted using the Kaplan–Meier method and comparisons between groups were made using the log-rank test using GraphPad Prism v 5.03

*S. suis* produces a secreted membrane damaging toxin, suilysin suggested which is to contribute to virulence [41]. An allelic replacement insertion mutant of *S. suis* strain P1/7 lacking functional *sly*, (Δ*sly*), is not haemolytic and unlike the wild type P1/7 does not efficiently kill J774.2 cellsin vitro [40]. Furthermore, the deletion of suilysin mutation prevented *S. suis* from killing mice via the intraperitoneal route of infection [40]. In pig infection models, the mean survival times of the wild type P1/7 and the suilysin mutant Δ*sly* were not significantly different [42]. In *G. mellonella* infection with 10^7^ CFU/ml of the Δ*sly* mutant showed slightly more than 2 times attenuated virulence 24 p.i. compared to the wild type P1/7 (Fig. 4). However, 48 to 144 h p.i. the virulence of the Δ*sly* mutant was attenuated more than 1.5 fold compared to the wild-type strain P1/7. The attenuated virulence of the Δ*sly* mutant in *G. mellonella* larvae compared to the wild-type stain P/7 is in agreement with the effects observed in J774.2 cells in vitro and in the interperitoneal mice infection model.

## Discussion

The use of insect larvae for virulence testing of pathogens is not regulated by legislation and represents an alternative to experimental infection of rodents or pigs. Alternative infection models are important in order to comply with the efforts to reduce, refine, and replace animal experiments (the 3Rs). *G. mellonella* larvae have been used as infection models for other important pathogens [29] and to study the efficacy of antibacterial and antifungal therapies [28, 43, 38] due to the general similarities between the wax worm innate immune system and the innate immune response of higher organisms [33]. As an infection model *G. mellonella* offers a number of technical advantages over mammalian infection models (e.g. high-throughput, ease of handling, simple housing requirements, the possibility to purchase a large number of larvae at a relatively low price, no legal or ethical issues etc.). Importantly, experiments with *G. mellonella* larvae unlike experiments with other non-mammalian models (e.g. zebrafish larvae, *C. elegans*, or *D. melanogaster*) can be performed at the body temperature of the mammalian natural host, 37 °C. The immune system of insects, including *G. mellonella* larvae, is very similar to the innate immune response of mammals [33]. Therefore, results obtained using insects can serve as a starting point to study pathogenesis and generate hypotheses to be further tested in vertebrate models.

The present study aimed to validate *G. mellonella* larvae as an alternative model to investigate the virulence of *S. suis. S. suis* is an important zoonotic pathogen and significant cause of meningitis and sepsis in pigs and humans [6]. Clinical isolates of S*. suis* were shown to kill *G. mellonella* larvae in a dose-dependent manner. Furthermore, similar to infection with other pathogens, infection of *G. mellonella* larvae with virulent *S. suis* strains triggered melanisation (Additional file 1: Figure S2). Melanisaiton is part of the immune response in *G. mellonella* considered a key step of the defense against a range of pathogens [29]. Melanin is deposited around microbes within the haemolymph and is thought to facilitate pathogen killing. The strains with greater reported virulence in pigs were also more virulent in *G. mellonella* supporting the predictive value of the *G. mellonella* infection model. The correlation in virulence of *S. suis* isolates in pigs and *G. mellonella* was demonstrated using published virulence data, i.e. no pigs were required for the experiments reported here [35]. Additionally, we report on the virulence of several *S. suis* strains that have not been studied in pigs or rodent infection models. Live strains of *S. suis* were required to significantly increase larval mortality, the exception being strains 8039 and 5213 which still increased larval mortality when heat-killed prior to injection at the highest dose. Nevertheless, the effect of larval survival infection with 8039 or 5213 is comparable to the effect of the weakly virulent serotype 2 S735 or the non-capsulated serotype 2 mutant cpsΔEF.

The bacteria-free culture supernatants of *S. suis* did not induce significant death of larvae compared to the control except in the case of the serotype 9 strain 7709, which was among the most virulent strains tested in the *Galleria* infection model (Additional file 1: Figure S1, and Table S1). This might be due to higher expression of suilysin, the pore-forming toxin produced by most pathogenic strains of *S. suis*, which is carbon catabolite repressed by glucose when grown in vitro [44].

## Conclusion

The use of invertebrate hosts such as *G. mellonella*, *D. melanogaster* and *C. elegans* to study microbial pathogenesis and host responses have contributed substantially to biomedical research over the last decades. In this study, we describe the utility of *G. mellonella* larvae to study the virulence of the zoonotic pathogen *S. suis*. Relatively little is known about the pathogenic mechanisms of *S. suis*. Therefore, the infection model reported here serves as a useful starting point for assessing the strain virulence or the impact of genetic mutations on virulence of *S. suis*. Interestingly, a good correlation between the pathogenicity of *S. suis* in *G. mellonella* and the virulence observed in pigs and humans, and in other infection models such as zebrafish larval infection model and mice infection model was observed. Furthermore, the infection model described here also has the potential to be used to evaluate novel therapies for in vivo activity against *S. suis* and help prioritise candidates for testing in mammalian models.

## Additional file

Additional file 1: Figure S1. LD50s of tested strains. **Figure S2.** Infection with *S. suis* triggers melanisation in *G. mellonella* larvae. **Figure S3.** Effect of cell-free supernatant and heat-inactivated inocula on *G. mellonella* larvae survivial. **Table S1.** LD50s of tested strains. (DOC 2250 kb).

## Abbreviations

3Rs: Replacement, reduction, and refinement of animal experiments
AV: Avirulent
CFU: Colony forming units
CNS: Central nervous system
MHB: Mueller Hinton broth
p.i.: Post-infection
PBS: Phosphate buffered saline
TCS: Two-component system
V: Virulent
WV: Weakly virulent
