# Supplementary material for: Evaluation of Galleria mellonella larvae for studying the virulence of Streptococcus suis
Source: BMC Microbiol. 2016 Dec 15;16:291. doi: 10.1186/s12866-016-0905-2 (PMC5160000; doi:10.1186/s12866-016-0905-2)
Supplement: Additional file 1: Figure S1. — LD50s of tested strains. Figure S2. Infection with S. suis triggers melanisation in G. mellonella larvae. Figure S3. Effect of cell-free supernatant and heat-inactivated inocula on G. mellonella larvae survivial. Table S1. LD50s of tested strains. (DOC 2250 kb). [file 12866_2016_905_MOESM1_ESM.doc]

Supplementary material

Evaluation of *Galleria mellonella* larvae for studying the virulence of *Streptococcus suis*

Nadya Velikova*^a^, Kevin Kavanagh^b^, Jerry M. Wells^a^

^a^Host-microbe Interactomics Group, Department of Animal Sciences, Wageningen University, Wageningen, The Netherlands

^b^Department of Biology, Maynooth University, Co. Kildare, Ireland

Page 2-4 Supplementary Figures

Page 5 Supplementary Table


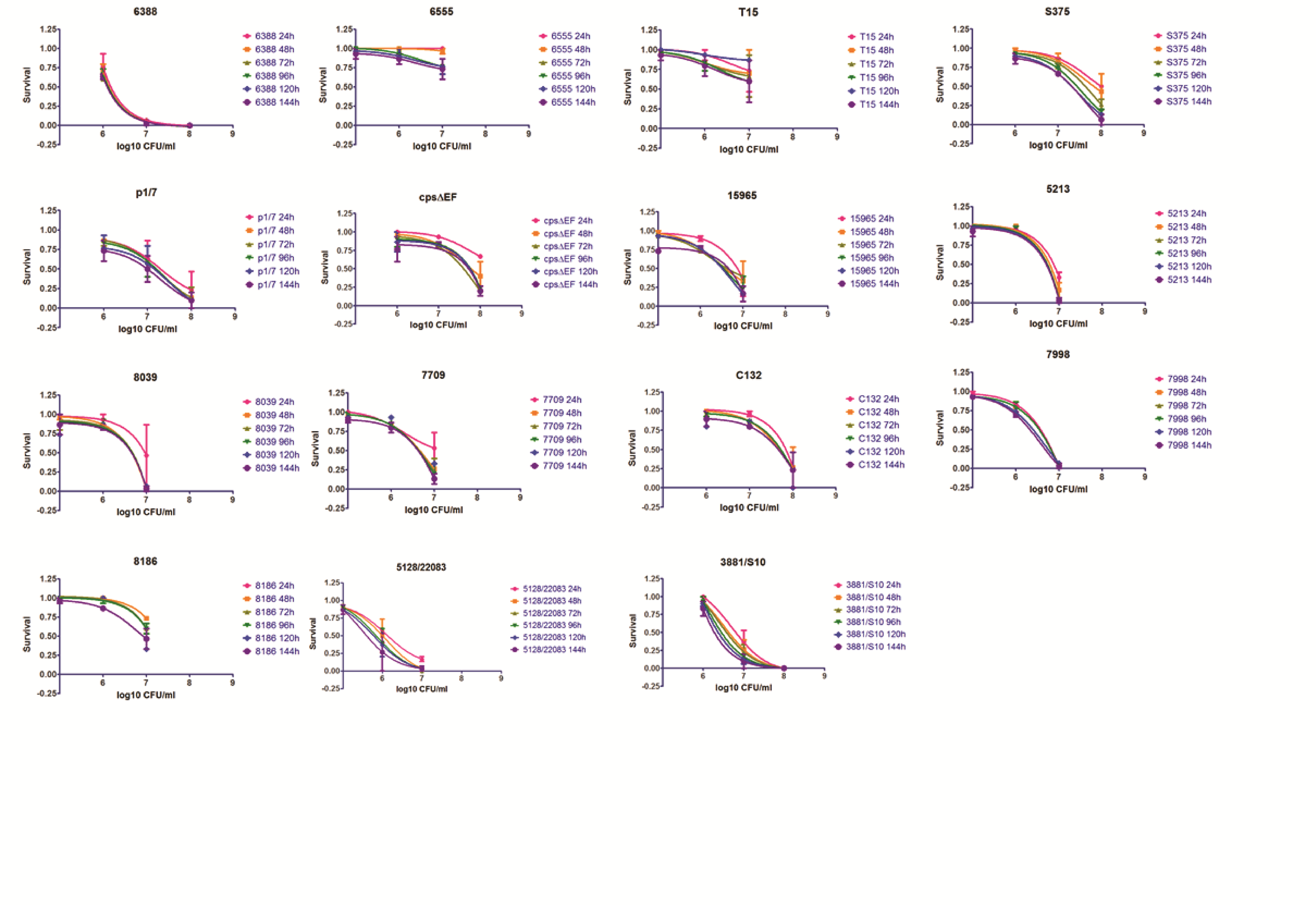


Figure S1. LD50s of tested strains. The lethal dose leading to 50% mortality (LD50) at different time points was determined by plotting the percent survived larvae following infection with different inocula, and non-linear curve fitting using GraphPad Prism v 5.03.


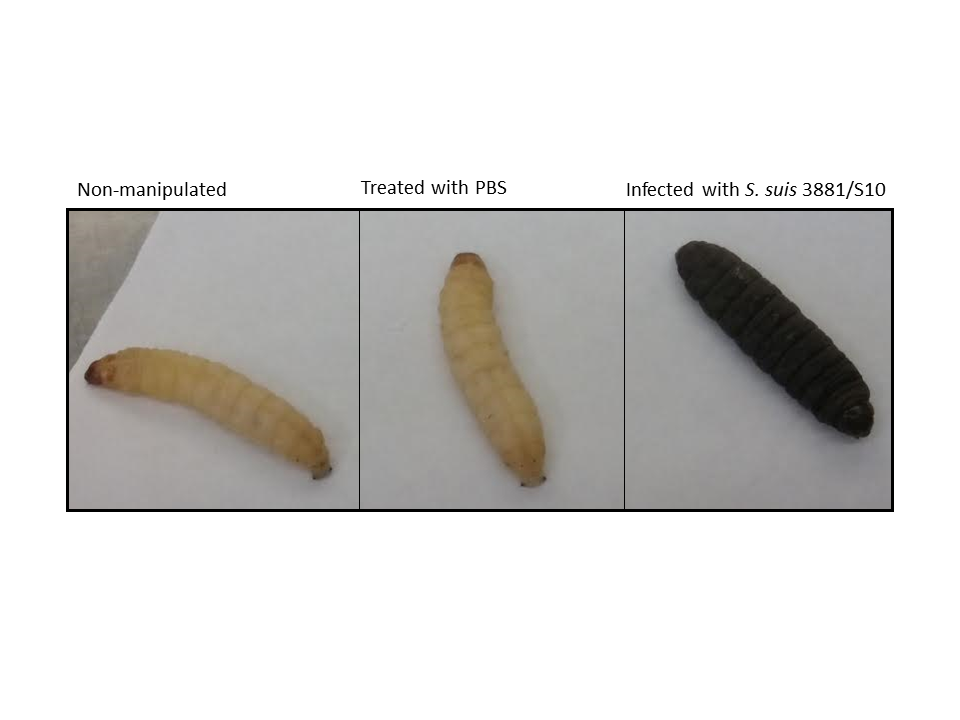


Figure S2. Infection with *S. suis* triggers melanisation in *G. mellonella* larvae


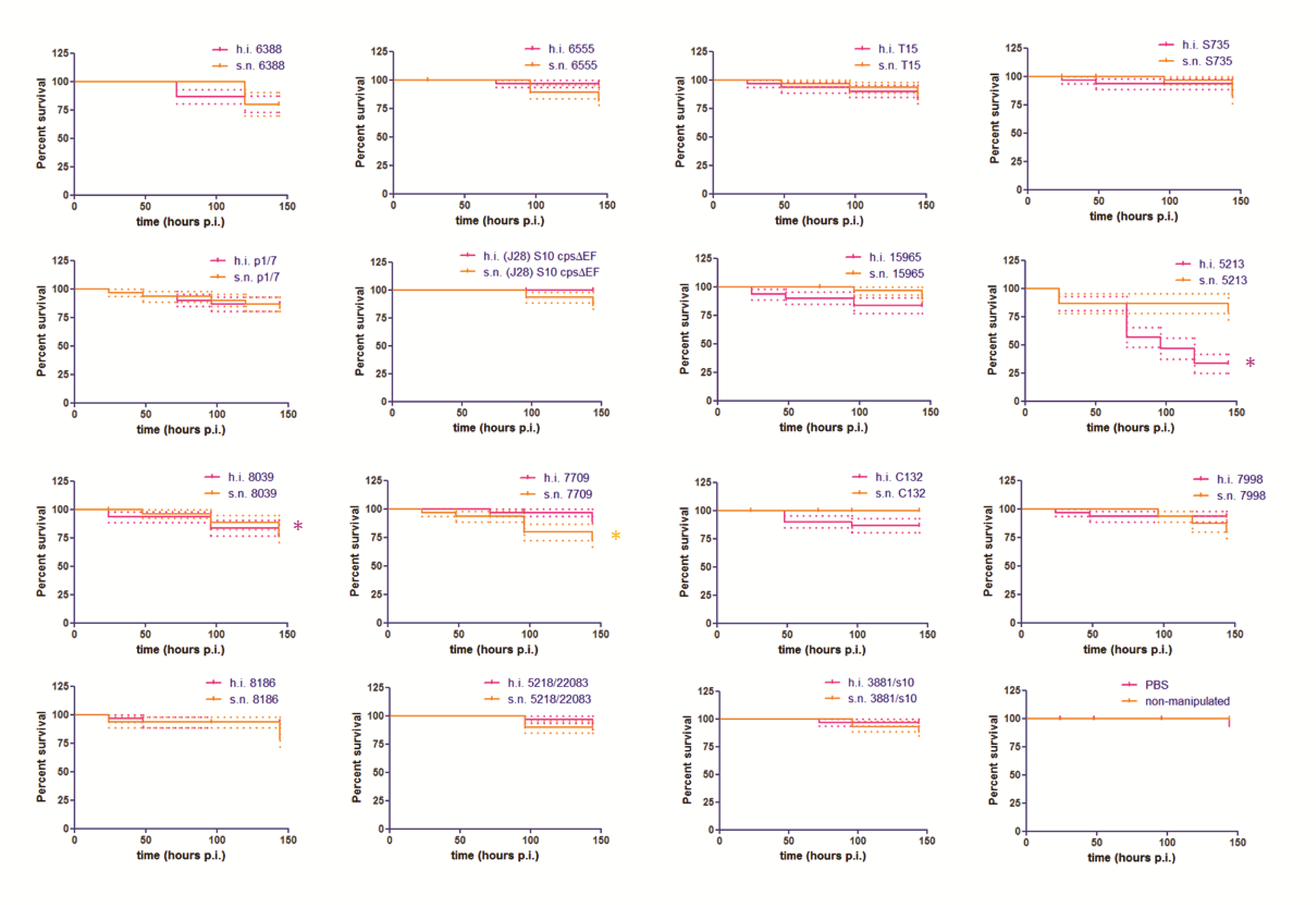


Figure S3. Effect of cell-free supernatant and heat-inactiated inocula on G. mellonella larvae survivial. In all but three strains live bacteria are needed for infection. The heat-inactivated 8039 and 5213 caused significant change in larval survival (p values of 0.0481 and < 0.0001, respectively) compared to the non-infection control (PBS). The cell-free supernatant of 7709 caused significant change in the larval survival (p value 0.0085) compared to the non-infection control.

Table S1. LD50s of tested strains. The lethal dose leading to 50% mortality (LD50) at different time points was determined by plotting the percent survived larvae following infection with different inocula, and non-linear curve fitting using GraphPad Prism v 5.03.

| **serotype** | **LD50 CFU/ml** | | | | | |
| --- | --- | --- | --- | --- | --- | --- |
|  | **24** | **48** | **72** | **96** | **120** | **144** |
| **6388** | 1.E+04 | 8.E+02 |  | 6.E+02 | 5.E+02 | 5.E+02 |
| **6555** | 1.E+06 | 1.E+11 |  | 3.E+06 | 2.E+06 | 2.E+06 |
| **5218/22083** | 1.E+06 | 1.E+06 |  | 7.E+05 | 6.E+05 | 3.E+05 |
| **7709** | 2.E+06 | 7.E+06 | 8.E+06 | 9.E+06 |  | 2.E+07 |
| **T15** | 4.E+06 | 1.E+06 | 1.E+06 | 2.E+06 | 1.E+06 | 2.E+06 |
| **3881/S10** | 5.E+06 | 3.E+06 | 2.E+06 | 1.E+06 | 8.E+05 | 4.E+05 |
| **7998** | 1.E+07 | 1.E+07 | 1.E+07 | 1.E+07 | 5.E+06 | 4.E+06 |
| **P1/7** | 2.E+07 | 2.E+07 | 2.E+07 | 3.E+07 | 3.E+07 | 2.E+07 |
| **15965** | 4.E+07 | 3.E+06 | 2.E+06 | 5.E+06 | 5.E+06 | 3.E+10 |
| **S375** | 6.E+07 | 4.E+07 | 7.E+07 | 4.E+07 | 3.E+07 | 4.E+07 |
| **cpsΔEF** | 7.E+07 | 5.E+07 | 8.E+07 | 9.E+08 | 8.E+10 | 8.E+10 |
| **8039** | 5.E+08 | 5.E+07 | 2.E+10 | 2.E+10 |  | 1.E+10 |
| **5213** | 4.E+10 | 4.E+10 | 4.E+10 | 4.E+10 | 4.E+10 | 4.E+10 |
| **C132** | 6.E+10 | 8.E+07 | 2.E+08 | 2.E+08 |  | 1.E+08 |
| **8186** | 6.E+10 | 6.E+10 | 2.E+10 | 2.E+09 |  | 7.E+06 |
